# A Doping Lattice of Aluminum and Copper with Accelerated Electron Transfer Process and Enhanced Reductive Degradation Performance

**DOI:** 10.1038/srep31797

**Published:** 2016-08-18

**Authors:** Lin Zhang, Xue Gao, Zhixuan Zhang, Mingbo Zhang, Yiqian Cheng, Jixin Su

**Affiliations:** 1 School of Environmental Science and Engineering, Shandong University, Jinan 250100, China

## Abstract

Treatment of azo dye effluents has received increasing concerns over the years due to their potential harms to natural environment and human health. The present study described the degrading ability of the as-synthesized crystalline Al-Cu alloys for removal of high-concentration Acid Scarlet 3R in alkaline aqueous solutions and its degradation mechanism. Al-Cu alloy particles with Al/Cu ratios 19:1 were successfully synthesized by high-energy mechanical milling. Characterization results showed that 10 h mechanical alloying process could lead to the formation of crystalline Al(Cu) solid solution. Batch experiment results confirmed the excellent ability of Al-Cu alloy particles for the degradation of 3R in aqueous solution. Under a certain condition ([Al-Cu]_0_ = 2 g/L, [3R]_0_ = 200 mg/L, [NaCl]_0_ = 25 g/L, initial pH = 10.9), the 3R could be completely degraded within only 3 min. It was also found that the degradation reaction followed zero-order kinetics model with respect to the initial dye concentration. The intermediate compounds were identified by UV-vis, FT-IR and HPLC-MS, and a pathway was proposed. Additionally, post-treatment Al-Cu alloy particles were characterized by SEM and TEM, and the results showed that the degradation might be attributed to the corrosion effect of Al-Cu alloys.

Nanoporous metals have attracted continuous and growing attentions in both commercial applications and theoretical studies including the application in catalysis, sensors, fuel cells, aerospace and automotive industry, owing to their outstanding features of large specific surface area and high catalytic activity at room temperature^1^,^2^,^3^,^4^,^5^. Dealloying is the most widely used method to prepare nanoporous metals with three dimensional ligament-pore structure due to the simplicity and high efficiency^6^,^7^,^8^. Actually, dealloying in aqueous solution is a kind of corrosion reaction wherein one active component from the crystalline alloy selectively dissolves in solution, leaving a three-dimensional nanoporous structure of the remaining alloys behind^9^. Moreover, there is a simultaneous electron transfer process between active component and noble component along with the dealloying reaction, which result in eletrochemical catalytic ability.

Among various nanoporous metals applications, using nanoporous alloys to treat dyes wastewater has intrigued great attention due to their excellent catalytic properties. It is known that azo dyes wastewater is one of the most difficult industrial wastewaters to treat since its strong color, high COD and low biodegradability properties^10^,^11^,^12^,^13^,^14^,^15^. Also, azo dyes have potential hazard to public health and environment, even at trace levels due to their toxic, carcinogenic and mutagenic effects^16^,^17^,^18^,^19^. Therefore, the effective treatment of azo dyes from wastewater is urgently required. At present, various methods have been used to treat azo dyes, including adsorption, flocculation, oxidation, reduction, photodegradation, biological method and so on^11^,^20^,^21^,^22^. However, all above methods have their defects, for example, oxidation methods such as Fenton and photo-catalyst are expensive; biological methods are time-consuming; flocculation and adsorption are ineffective^23^,^24^. For these reasons, proper methods for the treatment of azo dyes wastewater should be based on the combination of quick pre-treatment and advanced biological treatment. In recent years, various alloy materials have emerged as promising catalysts or pre-treatment method for the removal of dyes from aqueous solution, which could convert organic molecules into simpler molecules and enhance the degradability of the azo dyes wastewater^25^,^26^,^27^,^28^,^29^,^30^,^31^,^32^. However, less report is presented so far to evaluate the degradation mechanism of dyes using alloys and most studies chose nanoporous alloys as catalyst. Therefore, further study is necessary to find out the relation between the dyes degradation mechanism and the dealloying process of alloys.

The purposes of this work are to investigate the relation between the corrosion reaction and degradation mechanism thorough the morphological change of crystalline alloy and its degrading ability to removal azo dyes from aqueous solution, also provide an effective and simple strategy for the pretreatment of azo dye wastewater. For this purpose, we synthesized Al-Cu alloys with crystalline Al(Cu) solid solution by mechanical alloying process. Al-Cu, as one of the most important Al-base alloys, are wildly applied in automobile, aircraft construction and space technology due to its high strength-to-weight ratio and good mechanical properties^33^,^34^. Additionally, aluminum alloys are sensitive to pitting corrosion along with the electrochemical process in specific liquid media, which is considered as one of the major process for the generation of active radicals^35^. Acid Scarlet 3R, an azo dye used in textile and paper industries, was chosen as a model reactive azo dye to be degraded for batch experiments. The effects of initial pH and initial 3R concentration on the removal of 3R dyes were investigated. The morphological characteristics and corrosion behaviors of the Al-Cu alloy particles were studied using SEM and TEM techniques. The post-treatment products were identified in detail by UV–vis absorption spectrum, FTIR and HPLC–MS techniques. A possible degradation pathway and mechanism were also proposed.

## Results and Discussion

### Characterization of Al-Cu alloys

**Figure 1:**
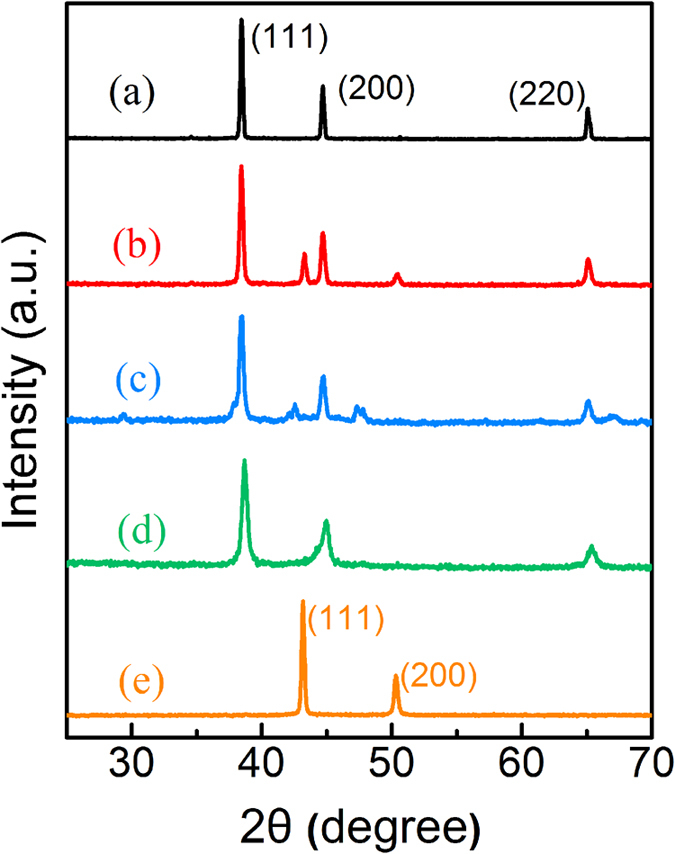
The XRD patterns of powders: (**a**) Al, (**b**) Al-Cu-1h, (**c**) Al-Cu-5h, (**d**) Al-Cu-10h, (**e**) Cu.

Figure 1 shows XRD patterns of the Al-Cu alloys powder after different milling times. The diffraction peaks of pure Al and pure Cu powder were presented in Fig. 1(a,e), respectively. It is clear that the intensity of Cu diffraction peak decreases and the width of Al peak increases progressively with the increase of milling time. As shown in Fig. 1(d), increasing milling time to 10 h led to the complete disappearance of Cu peaks, while the Al peak shifted from 2θ = 38.42° to 38.65°, Al peak shifted from 2θ = 44.69° to 44.97°, and Al (220) peak shifted from 2θ = 65.07° to 65.37°. The Al lattice parameter decreased from 4.053 to 4.033 Å. This can be attributed to the dissolution of Cu atom into Al lattice structure leads to the formation of Al(Cu) solid solution^36^.

**Figure 2:**
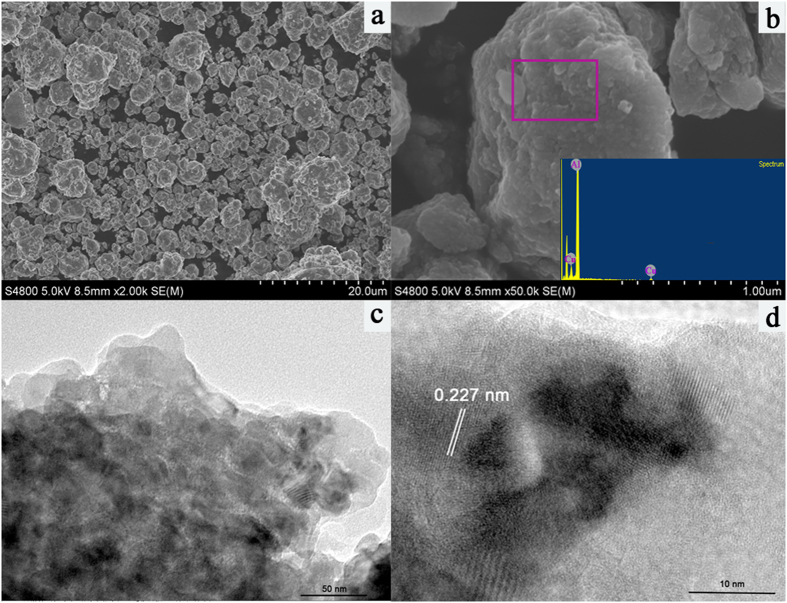
(**a**) Low magnification SEM images of Al-Cu-10h alloy particles; (**b**) High magnification SEM images of Al-Cu-10h alloy particles, insert: EDS spectrum (**c**) Low magnification TEM images of Al-Cu-10h alloy particles; (**d**) Surface microstructures of Al-Cu alloys.

Figure 2 shows the structure of Al(Cu) solid solution after MA 10 h. The morphology of the Al-Cu-10h alloy particles was shown in Fig. 2(a). It was evident that the particles are well dispersed without aggregation and sizes were relatively uniform. The diameters of particles were micrometer scale. As shown in Fig. 2(b), the surface morphology of powder particles was characterized by slight roughness. Simultaneously, the elementary composition was obtained by EDS to analyze the area in the frame of the SEM image (insert of Fig. 2(b)), revealing the presence of the Al and Cu elements, and the ratio of Al to Cu was approximately equal to 19:1, which did correspond to the needs of the alloy-synthesis experiment. Figure 2(c) shows the TEM images of the alloy powders. The metal particles were found to be uniformly dispersed. As shown in Fig. 2(d), the uniform lattice fringes further verified the single crystal nature of the superfine particles, and the lattice spacing of the particles was found to be 0.227 nm.

### Effect of operating parameters on degradation

#### Degradation of 3R under different systems

**Figure 3:**
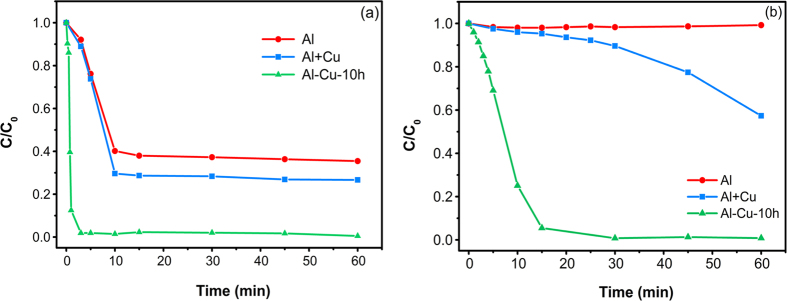
Degradation of 3R by different catalysts (Al, Al+Cu, Al-Cu) under acidic condition (**a**) and alkaline condition (**b**).

Figure 3 shows the degradation performances of 3R by the Al-Cu-10h particles, the pure Al powder and Al + Cu physical mixtures in different conditions. The experiments were carried out by adding 0.2 g different kinds of catalyst particles and 2.5 g of NaCl into 100 mL of the 3R aqueous solution ([Catalysts]_0_ = 2 g/L, [3R]_0_ = 50 mg/L, [NaCl]_0_ = 25 g/L), and the initial pH value of the 3R aqueous solution was adjusted to 2 (Fig. 3a) and 10.9 (Fig. 3b) by H_2_SO_4_ and Na_2_CO_3_, respectively.

As can be seen from Fig. 3a, little degradation of 3R was observed when adding into the 3R solution with pure Al power. The degradation efficiency was 40% after 60 min reaction with Al + Cu physical mixtures. The highest degradation efficiency was observed when adding with Al-Cu alloy particles by contrast with Al powder and Al + Cu mixtures.

In the alkaline condition, Fig. 3b showed the similar results with the acid condition experiment, the 3R degradation capacity was in the order of Al-Cu alloy particles >Al + Cu physical mixtures > pure Al powder. Besides, 100% of the 3R was degraded within only 3 min when Al-Cu-10h alloy particles was applied.

### Effect of initial pH

**Figure 4:**
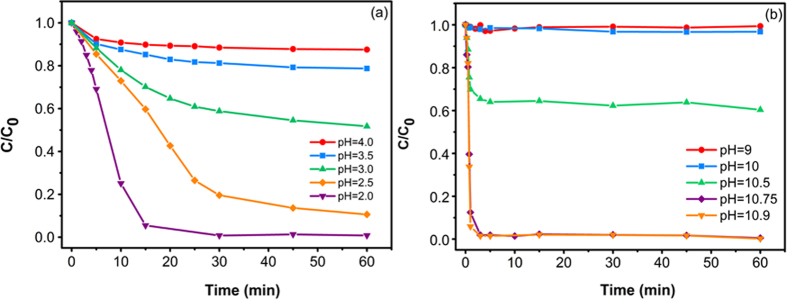
Effect of initial pH value on the degradation of 3R over Al-Cu-10h alloy particles under acidic condition (**a**) and alkaline condition (**b**).

In azo dye treatment systems, the pH value has been considered as one of the important factors^18^,^37^. In this work, the effect of initial solution pH on the degradation of 3R by Al-Cu alloys was studied both in acid pH (2–4) and alkaline pH (9–10.9). The rest of the operating parameters for the two experimental systems were the same ([Al-Cu-10h]_0_ = 2 g/L, [3R]_0_ = 50 mg/L, [NaCl]_0_ = 25 g/L). The results were shown in Fig. 4.

It can be seen from Fig. 4a that the degradation efficiency increased with decreasing pH value under the acid condition, and the maximum efficiency was observed at pH value equals 2 (~99% efficiency in 30 min). This phenomenon may be ascribed to the redox reactions (Eqs (1, 2, 3)) in acid solution. The increase in the concentration of H^+^ could significantly improve the anode reduction process (Eq. 2), then the active group [H] will attack azo dye molecules and thereby increase the degradation efficiency.


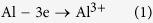



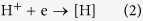



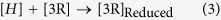


As shown in Fig. 4b, under the alkaline condition, the degradation efficiency of 3R increased with the increase of pH value and the maximum efficiency was observed when pH value was 10.9 (~100% efficiency in 3 min). Moreover, Al(Cu) solid solution structure could constitute a typical micro-cell or even nano-cell reaction system in alkaline solution. During the degradation process, Al as anode will lose electrons and react with OH^−^ to from Al(OH)_3_ and Al(OH)_4_^−^ (Eqs (4) and (5)); meanwhile, water molecules could absorb electrons to form active group [H] on the active sites, which will attack azo dye molecules and then hydrogenation cracking them. Besides, since 3R molecules are negatively charged, the existence of positive charged aluminum atom, Al^3+^ and Al(OH)_3_ are favorable for the adsorption of 3R molecules onto the Al-Cu alloy particles surface. However, at low pH value, a large number of Al(OH)_3_ precipitate will adhere on the surface of Al-Cu alloys and thus prevent the degradation of the 3R, but with increasing of pH value, the increased OH^−^ concentration would lead Al(OH)_3_ precipitate turn to dissolvable [Al(OH)_4_]^−^, which is benefit to improve the degradation efficiency.


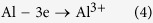



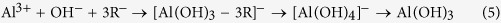



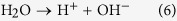



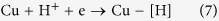



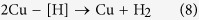



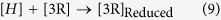


In short, alkaline condition is more suitable for the degradation of 3R solution by using Al-Cu alloys system. This result has a very practical significance because there is no need to add any acid into the actual industrial textile alkaline wastewater before the treatment.

### Effect of initial 3R concentrations

**Figure 5:**
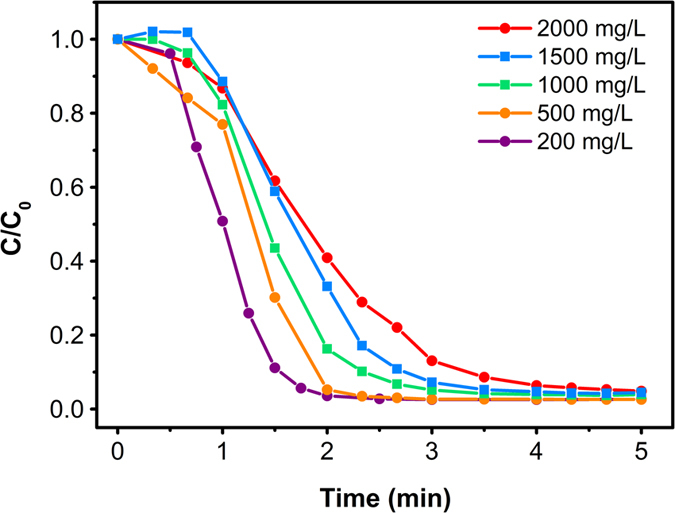
Effect of initial dye concentration on the degradation of 3R.

The effect of initial dye concentration on the degradation efficiency is a key factor in investigating the kinetics of the reaction. In order to evaluate the effect of dye concentration, The initial concentrations of 3R was selected in the range of 200 mg/L to 2500 mg/L, which was much higher than the treatment range of AOPs, also the other experimental parameters were kept constant ([Al-Cu-10h]_0_ = 0.2 g, [NaCl]_0_ = 2.5 g, [Na_2_CO_3_]_0_ = 0.1 g). As shown in Fig. 5, the degradation efficiency decreased with the increasing initial 3R concentration. Generally, the degradation of 3R in Al-Cu alloys system is a heterogeneous reaction, which involves adsorption of 3R on the reactive sites of Al-Cu particles, degradation reaction, and release of the degradation products. Increasing the initial concentration of 3R would lead to the competitive adsorption among the 3R molecules on the active site. Moreover, higher initial concentration of 3R would generate more intermediates, which would also compete with 3R molecules. In other words, increasing initial concentration of 3R would decrease the degradation efficiency to some extent. Nevertheless, due to the rapid production of active [H] on the surface active site, all degradation reactions reached their equilibrium within only 10 min, indicating that the Al-Cu alloys system is applicable to a wide range of initial dye concentration.

### Kinetic study

The reaction order was determined by using the relationship between reaction order and half-time (Eq. (10)) to fit the degradation reaction in first 3 min.


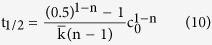


where t_1/2_ represents the half-life (s), C_0_ is the initial dye concentration (mg/L), k is the reaction rate coefficient (mg•L^−1^s^−1^), and n is the reaction order.

**Figure 6:**
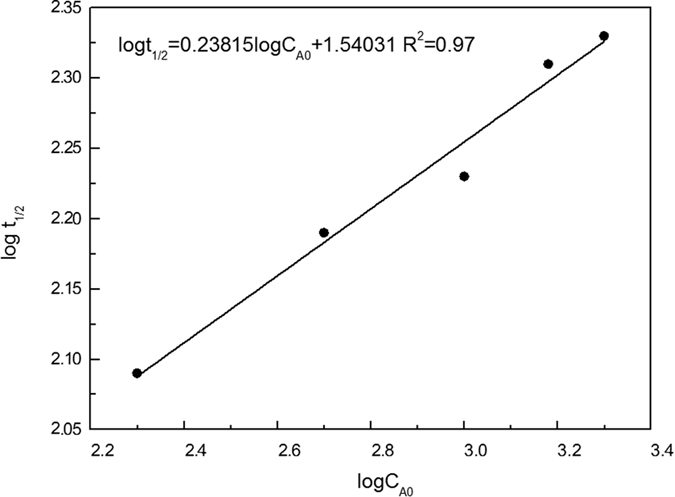
Relationship between initial dye concentration (C_0_) and half-time (t_1/2_) under the treatment of Al-Cu alloys.

**Figure 7:**
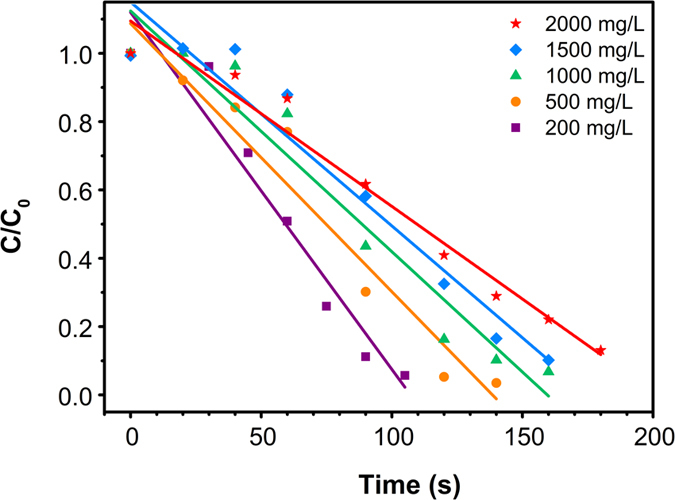
Kinetics of the degradation of 3R for different initial dye concentrations.

**Figure 8:**
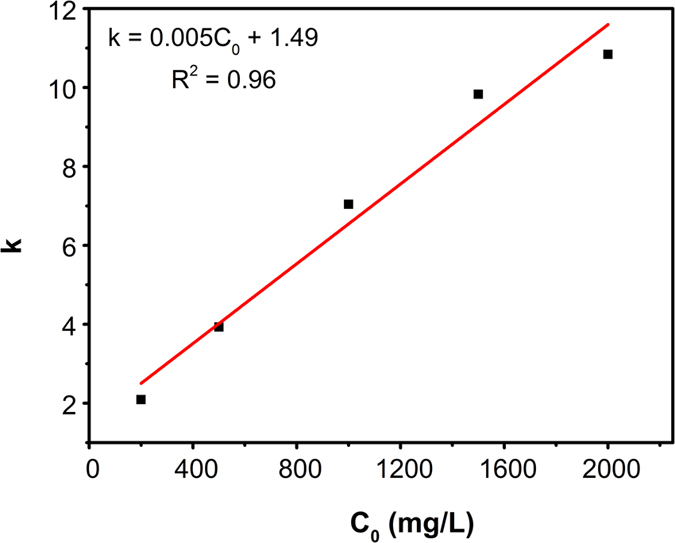
Relationship between k and C_0_.

As shown in Fig. 6, it was clear that the degradation of 3R with different initial concentrations (200–2500 mg/L) all followed zero-order kinetics. In the experiment, linear fitting could be observed in every experimental condition (Fig. 7), and the kinetic equation parameters are listed in Table 1. Additionally, the relationship between reaction rate constant (k) and initial concentration (C_0_) were shown in Fig. 8.

According to the traditional zero-order reaction, rate constant k is not changing with the change of the initial concentration. However, in this work, rate constant k increased from 2.09 to 12.17 s^−1^ when the initial concentration of 3R increased from 200 to 2500 mg/L. Generally, the mass-transfer of dye molecules and degradation intermediates between the solution phase and the surface of Al-Cu alloy particles would become the main limiting factor when the initial dye concentration was extremely high. However, in this Al-Cu alloys system, quick transfer of electrons from aluminum to copper could produce a large number of [H] around the active site, thereby promoting the generation of H_2_ in a very short time. A large amount of H2 will induce hydrogen spillover effect, and then cause a nano-flow around the copper active sites, which could transport the original 3R molecules and degradation products between liquid phase and active site surface. Therefore, the diffusion resistance result from increased degradation products could be avoided under hydrogen spillover effect.

### Identification of intermediates and degradation pathway

#### UV-Vis/FTIR spectral analysis

**Figure 9:**
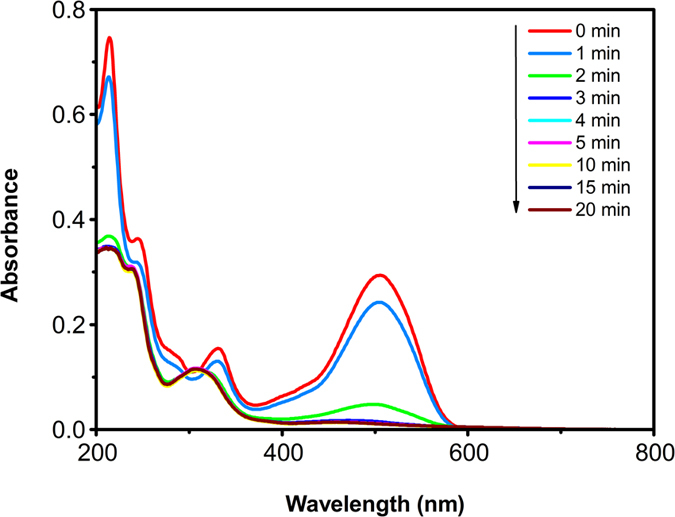
UV−vis absorption spectra changes of an AO7 aqueous solution during Al-Cu alloys treatment.

The changes in the absorption spectra of 3R in aqueous solution during the degradation process under a certain condition ([3R] = 1000 mg·L^−1^, [Al-Cu-10h] = 0.2 g·L^−1^, [NaCl] = 25 g·L^−1^, initial pH = 10.75) are shown in Fig. 9. The original absorption of dye solution mainly consisted of three peaks, it was clear that the absorbance in the visible region at 503 nm is attributed to the n-π^*^ transition of the –N=N– group, and the absorbance peaks at 214 and 331 nm were attributed to the π-π^*^ transition related to the benzene and naphthalene ring attached to the -N=N- group in the dye molecule^38^,^39^,^40^.

After 3 min treatment, it can be seen that all three main peaks (214, 331, and 503 nm) of Acid Scarlet 3R were almost removed completely. Meanwhile, two new peaks (209 and 307 nm) were generated in the UV-Vis spectrum. The result indicated that the azo groups (503nm) could be decomposed completely. Moreover, the two new peaks located 209 and 307 nm may result in the blue-shift effect of the benzene and naphthalene ring when the azo group was broken.

**Figure 10:**
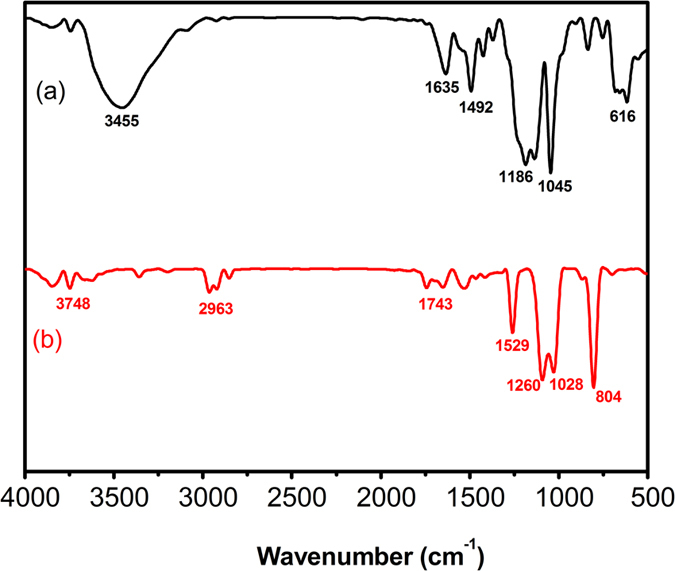
FTIR absorption spectra of 3R before and after treatment by Al-Cu-10h alloy particles: (**a**) Acid Scarlet 3R without treatment; (**b**) Acid Scarlet 3R after treatment.

The FTIR spectra of 3R before and after degradation were shown in Fig. 10. Curve a represented the FTIR adsorption spectrum of original 3R sample. The peak at 3455 cm^−1^ was attributed to the O-H stretching vibration, the peak at 1635 cm^−1^ was assigned to the azo bond (-N=N-). The peak at 1492 cm^−1^ was attributed to the C=C aromatic skeletal vibrations. The bands at 1045 and 1186 cm^−1^ were attributed to C-OH stretching vibration, while the peak at 616 cm^−1^ was characteristic of sulfonic group^41^,^42^,^43^.

Curve b showed the FTIR adsorption spectrum of 3R after 20 min treatment by Al-Cu alloy particles ([3R] = 1000 mg·L^−1^, [Al-Cu-10h] = 0.2 g·L^−1^, [NaCl] = 25 g·L^−1^, initial pH = 10.75). It is clear that the characteristic peaks of the azo bond (-N=N-, at 1635 cm^−1^) and the naphthalene ring (C=C aromatic skeletal vibration, at 1400–1600 cm^−1^) of 3R decreased remarkably. Meanwhile, several new peaks were observed after the treatment, the peak at 2963 cm^−1^ was assigned to the skeletal vibration of benzene ring. The peaks located 1742 cm^−1^, 1528 cm^−1^ and 1260 cm^−1^ were attributed to the stretching vibration of bond C=C, N-H and C-N, respectively. Additionally, the presence of peak at 1028 cm^−1^ due to the coupling between the benzene mode and stretching vibration of –SO_3_, and the peak at 804 cm^−1^ was linked to skeletal vibration of -CH_3_^41^,^43^,^44^,^45^. The results indicated that the azo bond and the naphthalene ring are broken by using Al-Cu alloy particles and resulting in the formation of amino and alkyl compounds.

#### HPLC-MS analysis

**Figure 11:**
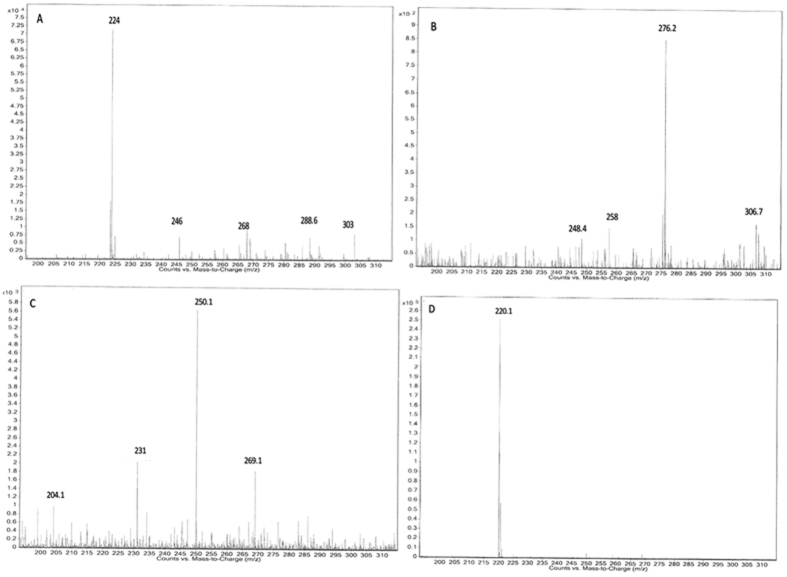
HPLC-MS analysis of 3R degradation products. (**A**) t = 4.31 min; (**B**) t = 4.91 min; (**C**) t = 5.50 min; (**D**) t = 6.20 min.

**Figure 12:**
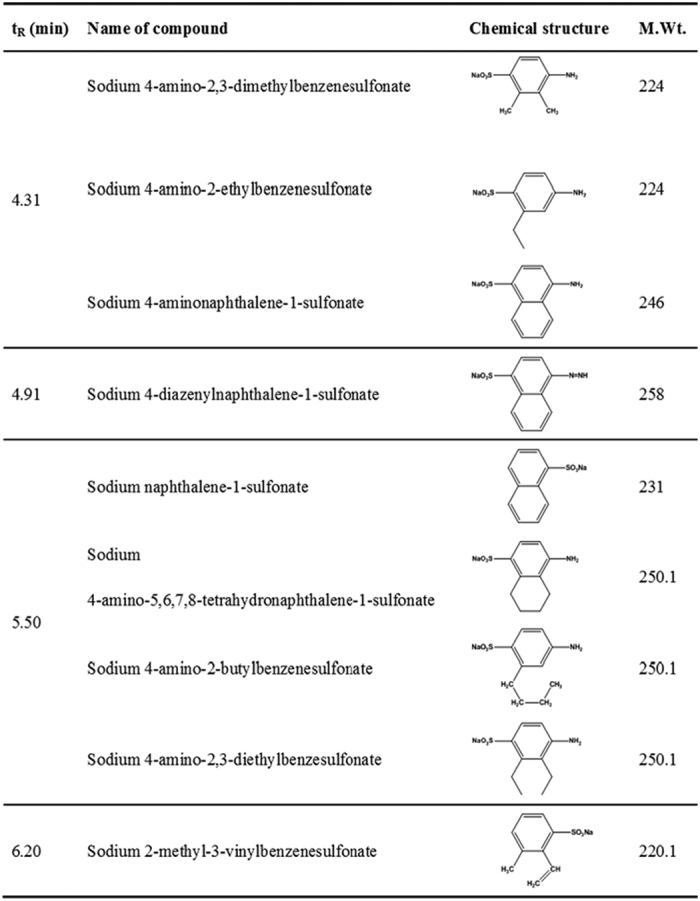
Proposed chemical structures of 3R degradation products identified by HPLC–MS.

**Figure 13:**
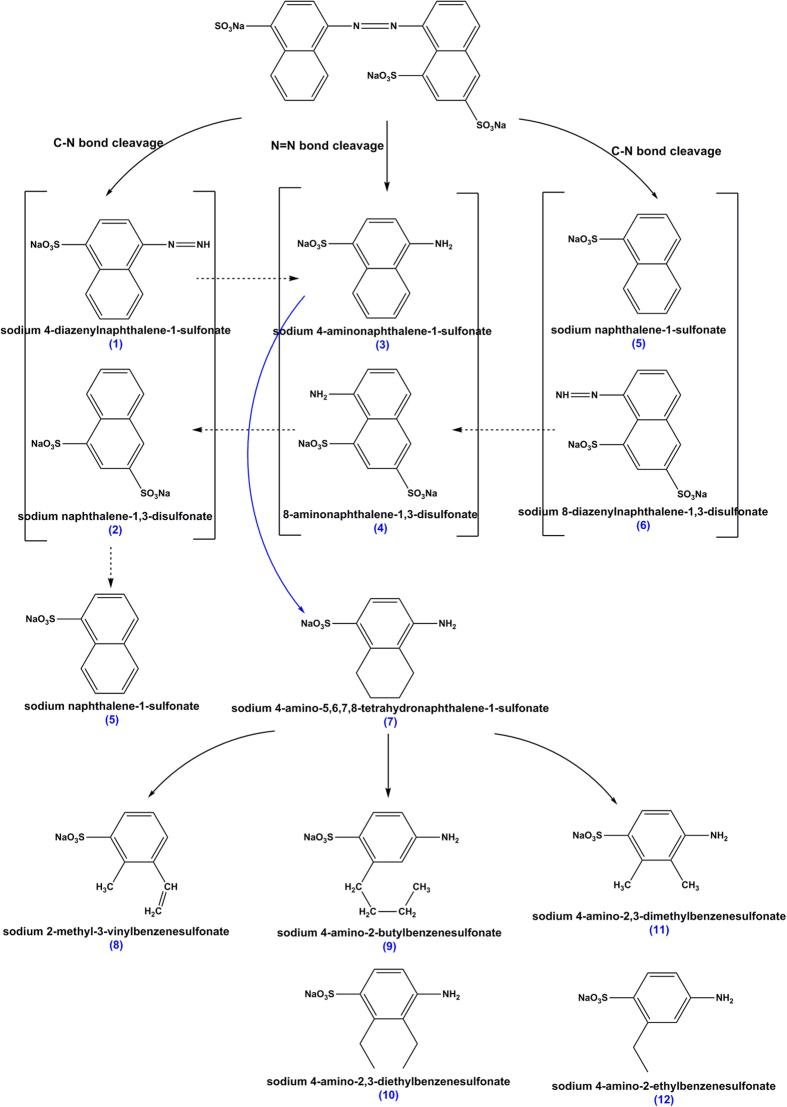
A possible pathway for the degradation of Acid Scarlet 3R in Al-Cu alloys system.

In order to better understand the degradation of Acid Scarlet 3R by Al-Cu alloys, HPLC-MS study was performed ([3R]_0_ = 1000 mg·L^−1^, [Al-Cu-10h]_0_ = 0.2 g·L^−1^, [NaCl]_0_ = 25 g·L^−1^, initial pH = 10.75, treatment time = 20 min). Figure 11 showed the mass spectra of degradation products identified by the HPLC-MS method. The proposed molecular formulas and m/z value were summarized in Fig. 12. On the basis of identified products, a possible pathway for 3R degradation was proposed in Fig. 13. In this degradation pathway, all aromtic by-products are assumed to be reduced by [H]. As shown in Fig. 13, degradation of 3R was firstly initiated by the break of chromophore center (-N=N-) leading to the decolorization of the solution and the formation of (3) and (4). At the same time, another two routes proceed through the break of C-N bond, which leads to the formation of compound (1), (2), (5), (6). The consecutive hydroxylation of compound (3) resulted in the formation of compounds (7). Afterwards, further hydrogenation of compound (7) with ring opening leads to the formation of benzenic compounds (8–12). The supposed degradation pathway was in agreement with the above UV-vis and FTIR analysis.

#### Characterization of post-treatment Al-Cu alloys

**Figure 14:**
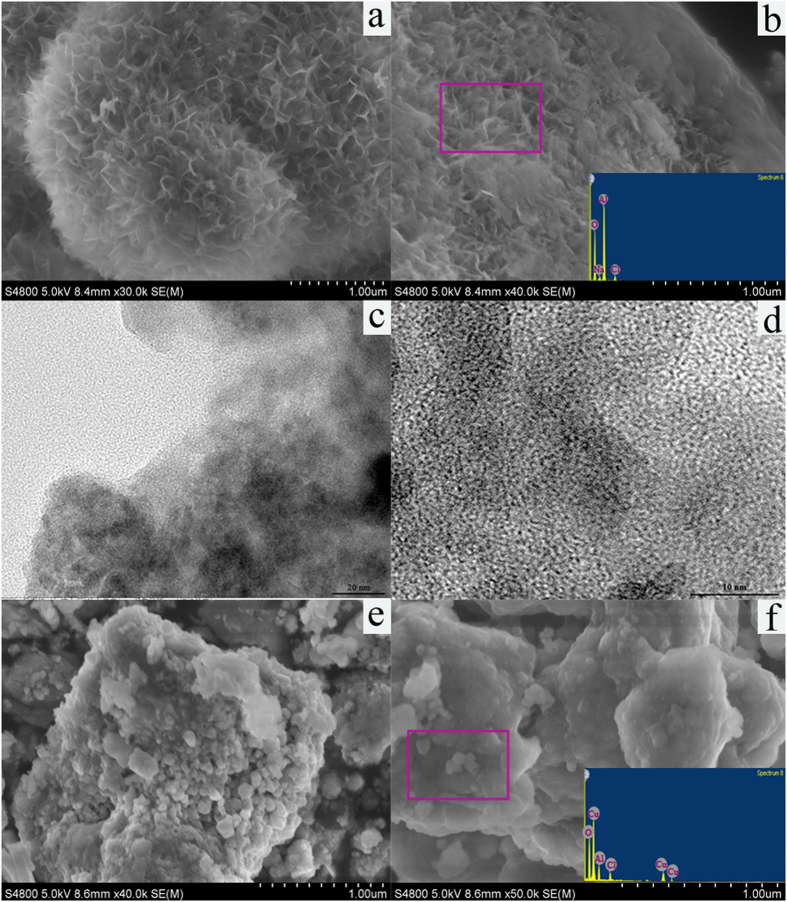
SEM and TEM images of Al-Cu-10h alloy particles after the decolorization reaction (**a–d**) and after the strong corrosion reaction (**e,f**).

To clarify the degradation mechanism associated with the corrosion of Al-Cu alloys, their corrosion morphologies should be clearly distinguished. The surface morphology of the Al-Cu-10h alloy particles after the degradation process was shown in Fig. 14. The result showed that the part of surface compactly was covered by flower-like products. From EDS results (inset of Fig. 14b), the flower-like products were some flocculent materials mainly composed of the Al element. Furthermore, according to the results from TEM (Fig. 14d), many corrosion pits were observed on the alloys surface, which indicating the pitting corrosion on alloy particles took place during the process of 3R degradation in alkaline medium. It has been acknowledged that corrosion of Al-based alloy depends on the damage of passive film like Al_2_O_3_. Also, pitting corrosion is thought to be one of the principal mechanisms for the degradation of the 3R. When the local pH value around the Al-Cu alloy particles increased, the stable Al(OH)_3_ formed by the reaction between Al^3+^ ions and hydroxide ions (OH^−^).

Afterwards, we designed the strong corrosion experiment for the Al-Cu alloys ([Al-Cu-10h]_0_ = 2 g/L, [3R]_0_ = 50 mg/L, [NaCl]_0_ = 25 g/L, initial pH = 14, treatment time = 30 min). For the as-received alloy, the surface is seriously corroded and the corrosion pits become larger (Fig. 14e). Also, the EDS result (insert of Fig. 14f) showed that the ratio of the aluminum and copper dropped down under the strong corrosion environments. The results evidence the alloy corrosion was initiated by pitting corrosion, and with the degradation reaction, anodic Al constantly dissolve into the solution.

#### Degradation Mechanism

**Figure 15:**
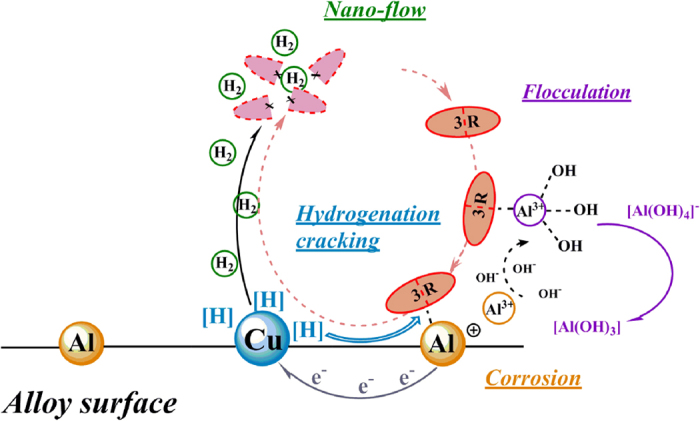
A proposed degradation mechanism of Acid Scarlet 3R in Al-Cu alloys system.

Based on the above batch experiments and characterization results, the degradation mechanism could be speculated as follows:

(1) The MA process led to the formation of crystalline Al(Cu) solid solution, which constitute micro-cell system under alkaline condition. In this system, the electrons derived from aluminum could attack the electron acceptors like azo group and thus reducing them, meanwhile, anodic copper could accept electrons and react with water to form active group [H] which also have ability to reduce 3R molecules^46^,^47^. (2) At the same time, a large amount of H_2_ was generated by the combination reaction of [H], which will cause nano-flow to accelerate the transformation between liquid phase and active site surface, and prevent the particles accumulation, remove the degradation products from alloy active site, thereby keeping the fresh surfaces and high reactivity of the particles. (3) The electrochemical corrosion reaction of the Al-Cu alloys in alkaline solution induces the formation of pitting corrosion, which enlarged the contacting areas of the 3R molecules and Al-Cu alloy particles, thus increasing the degradation efficiency. (4) At alkaline pH, the alloy particles will surrounded by positively charged Al atom and Al^3+^, since the dye molecules with sulfuric groups are negatively charged, the electrostatic interactions and flocculation between the Al-Cu alloy particles surface and dye molecules may occur, which leads to greater absorption of dye and increased degradation efficiency. (5) As a result of all above reactions, the degradation of 3R was accorded with the zero-order kinetic model, which ensured the reaction performed quickly and the application of the system in treatment of azo dyes with high initial concentration. Moreover, the fundamental reaction mechanism was presented in Fig. 15.

## Conclusion

In summary, the study provided a potential method for the pre-treatment of hardly degradable azo dye wastewater. It was demonstrated that as-synthesized crystalline Al-Cu alloys was a high activity catalyst for the degradation of Acidic Scarlet 3R in alkaline aqueous solution. Al-Cu alloys was prepared by mechanical alloying and the characterization results showed the formation of crystalline Al(Cu) solid solution. Batch studies demonstrated that the degradation efficiency of 3R increased with increasing acidity/alkalinity and decreased with increasing initial 3R concentration. Also the degradation process was found to fit zero-order kinetic model with respect to the dye concentration. The SEM and TEM analysis of the Al-Cu particles after degradation indicated the pitting corrosion occurred on the surface of Al-Cu alloy particles which was the principal mechanism for the degradation of azo dye. Afterwards, the post-treatment products were characterized using UV-vis, FTIR and HPLC-MS studies. As a result, amino and alkyl compounds were indentified, and a pathway was proposed for the degradation of 3R.

## Experimental

### Materials and Reagents

Acid Scarlet 3R is of industrial grade and used without further purification. Copper powder (200 mesh, 98.0% purity), aluminum powders (200 mesh, 99.0% purity) and all the other chemical reagents are of analytical grade. All solutions were prepared with deionized water.

### Synthesis of Al-Cu alloys

Mechanical alloying (MA) of Al-Cu alloys was performed under nitrogen atmosphere using a GN-2 high-energy ball-milling machine (Shanghai Hua Yan Equipment Co., Ltd.) and the molar ratio of the Al to Cu was 19:1. The stainless steel balls of 6, 10 and 12 mm diameters were used as milling medium, with a mass ratio of 4:1:1, and value of ball-to-powder weight ratio was 15:1. Absolute alcohol (8 wt% of powders) was used as process control agent to prevent excessive welding of powders. The rotation speed was selected as 600rpm. After 1 h, 5 h and 10 h of mechanical milling, the powders were collected and labeled as Al-Cu-1h, Al-Cu-5h, Al-Cu-10h, respectively.

### Batch experiments

Typically, the removal of the 3R aqueous solution by Al-Cu alloy particles was investigated thoroughly in batch experiments at room temperature. 3R aqueous solutions with different concentrations were prepared by simple dissolution in deionized water; meanwhile, a certain amount of NaCl was added into the solution to achieve high-salinity condition, which was similar to the sanity of actual industrial textile wastewater. In each experiment, 100 mL 3R aqueous solution, 0.2 g alloy particles and 2.5 g NaCl were added in a 500 mL beaker, and the mixed slurry was under vigorous stirring. At given time intervals, 5 mL of the aqueous solution was filtered through a Millipore filter (0.45 μm) to remove particles. Furthermore, the filtrates were then diluted to a suitable concentration range and analyzed by recording the absorbance of 3R using a UV-2550 UV-vis spectrophotometer.

### Analytical Methods

Phase identification of alloys was studied by X-ray diffraction (XRD, Rigaku D/Max-RA). The surface morphology and elementary composition was studied by Scanning electron microscopy (SEM with EDS, Hitachi/S3500). The size distribution and crystal structure was studied by transmission electron microscopy (TEM, JEM-100CXII) and high-resolution transmission electron microscopy (HRTEM, JEM-2100). The concentrations of 3R were quantified by a UV–vis spectrophotometry (UV-2550). FTIR spectra of the Al-Cu alloys catalysts before and after reaction were recorded as KBr pellets in the spectral range 4000–400 cm^−1^ on a Avatar 370 FTIR spectrometer at room temperature. The intermediates from the reduction of azo dyes molecules were analyzed by high-performance liquid chromatography-mass spectrometry (HPLC–MS) using a 1200HPLC/API4000MS.

The efficiency of the degradation is defined as


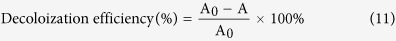


where the A_0_ and A are the absorbance of the sample at time 0 and t, respectively.

## Additional Information

**How to cite this article**: Zhang, L. *et al.* A Doping Lattice of Aluminum and Copper with Accelerated Electron Transfer Process and Enhanced Reductive Degradation Performance. *Sci. Rep.* **6**, 31797; doi: 10.1038/srep31797 (2016).

## Figures and Tables

**Table 1:** Zero-order kinetic parameters of X-3B degradation.

Concentration (mg/L)	Linear equation	k/s^−1^	R^2^
200	C = −2.09 t+213.95	2.09	0.93
500	C = −3.93 t+543.56	3.93	0.94
1000	C = −7.04 t+1123.02	7.04	0.94
1500	C = −9.83 t+1534.90	9.83	0.93
2000	C = −10.84 t+2180.62	10.84	0.96
