# Corrigendum: A Doping Lattice of Aluminum and Copper with Accelerated Electron Transfer Process and Enhanced Reductive Degradation Performance

**DOI:** 10.1038/srep46726

**Published:** 2017-04-21

**Authors:** Lin Zhang, Xue Gao, Zhixuan Zhang, Mingbo Zhang, Yiqian Cheng, Jixin Su

Scientific Reports 6: Article number: 31797; 10.1038/srep31797 published online: 08 18 2016; updated: 04 21 2017.

In this Article, the compounds in the titles of Table 1 and Figure 9 have been mislabelled.

The title of Table 1:

“Zero-order kinetic parameters of X-3B degradation”.

should read:

“Zero-order kinetic parameters of Scarlet 3R degradation”.

The title of Figure 9:

“UV–vis absorption spectra changes of an AO7 aqueous solution during Al-Cu alloys treatment”.

should read:

“UV–vis absorption spectra changes of a Scarlet 3R aqueous solution during Al-Cu alloys treatment”.

